# Understanding the physical activity promotion behaviours of podiatrists: a qualitative study

**DOI:** 10.1186/1757-1146-6-37

**Published:** 2013-09-09

**Authors:** Paul Crisford, Tania Winzenberg, Alison Venn, Verity Cleland

**Affiliations:** 1 Menzies Research Institute Tasmania, 17 Liverpool St, Hobart, TAS 7000, Australia

**Keywords:** Physical activity promotion, Podiatrist

## Abstract

**Background:**

Health professionals are encouraged to play a part in reducing the health risks of physical inactivity. Little is known of the physical activity promotion practice behaviours of podiatrists.

**Methods:**

We performed 20 semi-structured interviews with purposefully selected podiatrists to explore their physical activity promotion attitudes, beliefs, knowledge and practice. Transcribed interviews were coded using an iterative thematic approach to identify major themes and salient beliefs.

**Results:**

Overall, the participants had a positive attitude to physical activity promotion, considering it a normal part of their role. They saw their role as giving information, encouraging activity and making recommendations, however in practice they were less inclined to follow up on recommendations, monitor activity levels or document the process. Their approach was generally opportunistic, informal and unstructured and the content of assessment and promotion dependent upon the presenting patient’s condition. Advice tended to be tailored to the patient’s capabilities and interests. They considered there are opportunities to promote physical activity during regular consultations, however, were more likely to do so in patients with chronic diseases such as diabetes. Main barriers to physical activity promotion included unreceptive and unmotivated patients as well as a lack of time, skills and resources.

**Conclusions:**

Physical activity promotion appears feasible in podiatry practice in terms of opportunity and acceptability to practitioners, but there is scope for improvement. Strategies to improve promotion need to consider the major issues, barriers and opportunities as well as provide a more structured approach to physical activity promotion by podiatrists.

## Background

There is overwhelming evidence of the numerous benefits of engaging in regular physical activity [1-3]. Physical inactivity is linked to an increased risk of mortality and morbidity from a range of diseases and conditions [4]. However, in Australia, a national physical activity survey found that 66.9% of adults were either sedentary or had low levels of exercise [5].

The World Health Organisation emphasises that all health professionals should recognise that physical activity promotion can be used in the prevention and treatment of diseases and that their contact with patients provides an ideal opportunity to promote physical activity [6]. In Australia a governmental report suggested that health professionals of all types are potentially well placed to provide assessment, practical information, support and referral for individuals who may need assistance to get started, or to maintain regular physical activity [7].

There have been a number of studies that have looked at the factors associated with physical activity promotion by health professionals and these have primarily focused on their practice behaviors, knowledge, attitudes and beliefs. The majority of these studies observed general medical practitioners [8-11] with only a limited number of studies giving specific attention to other health professionals such as dietitians [12], nurses [8,13] pharmacists [14], physiotherapists [15] and clinical psychologists [16,17]. These studies have given insights into the practice behaviors and receptiveness to physical activity promotion of each profession along with the feasibility and practicality of physical activity promotion within each professional setting. The insights gained from these studies are thought to be beneficial in the implementation of effective change strategies [18].

While it is possible that these studies and their insights may have relevance to the podiatry profession, little is known about the podiatrists’ role in physical activity promotion. There is only limited information reporting the factors associated with the podiatrists’ role in health promotion [19] and even less regarding their role in physical activity promotion [20]. At present, clinic practice guidelines for promoting physical activity in the podiatry setting do not exist and furthermore, little is known about the extent to which podiatrists incorporate physical activity assessment and promotion into their clinical practice and the factors associated with it. It is possible that the podiatry setting provides an unexploited and undeveloped opportunity for physical activity promotion and podiatrists could play an important public health role.

The aim of this study, therefore, was to identify:

1) Podiatrists’ physical activity assessment and promotion practices.

2) The barriers and enablers facing podiatrists in physical activity assessment and promotion.

3) Podiatrists’ salient beliefs about and attitudes towards physical activity assessment and promotion and their role.

## Methods

We performed 20 semi-structured interviews with Tasmanian podiatrists purposefully selected to ensure a broad representation. The sampling frame was a list of Tasmanian practising podiatrists complete with contact details sourced from publicly available health practitioner registers [21], electronic [22] and local telephone directories [23] and through personal knowledge of one author (PC) (population n = 90). Podiatrists were selected in order to cover a range of demographics to facilitate collection of a diversity of views. Recruitment was by letter of invitation and non-responders were followed up with a phone call. All participants gave written consent and the interviews were carried out within the participant’s place of practice or alternatively at a place of their choosing. The interviews were carried out by a research assistant (CC) (n = 8) and by a clinical podiatrist (PC) (n = 12). Ethical approval was granted by the Human Research Ethics Committee Tasmania.

Semi-structured interviews were used because they are well suited for an exploratory study of the participant’s experiences and views. They allow the researcher and participant to engage in a dialogue in which initial questions are modified in the light of the participants’ responses and the researcher is able to probe interesting and important areas which may arise. This method enables the identification of detailed perceptions, opinions, beliefs, and attitudes of participants whilst allowing for flexibility of coverage and insights into novel areas [24]. Face to face interviews also have logistical advantages over focus groups, for example, in that they are more flexible as to location and timing, making it easier to accommodate the scheduling of data collection with busy health professionals.

The initial aim was to interview 20 podiatrists, with a view to continuing to interview further participants only if data saturation (no new themes were observed in the analysis, nor new data categories produced) was not achieved with this number of interviews. As data saturation was achieved, interviewing ceased after 20 interviews.

The Theory of Planned Behaviour (TPB) [25] theoretical model was used in the design of the interview schedule (Additional file 1: Table S1) to help identify and explain the beliefs, attitudes and behaviour in the promotion of physical activity by podiatrists. The TPB states that any given behaviour by professionals is influenced by the individual’s intentions to perform the specific behaviour and that these intentions are determined largely by attitudes toward the behaviour, perceived social norms, and perceived control related to the behaviour [25]. The TPB has been used to identify and predict healthcare practitioners’ behavioural intentions [26]. The development of the interview schedule was also influenced by a general overview of the literature and in particular some key papers [8,10,27,28]. Survey information was also collected about each podiatrist’s demographic and physical activity characteristics (Additional file 2) to allow us to check that we had in fact interviewed a diverse range of participants and to ascertain whether there were any obvious patterns of themes across different demographic attributes. The interview schedule and survey were piloted with two health professionals who were not part of the study. This was done to ensure a clear understanding of questions by both the interviewers and participants. Some minor modifications were made to ensure clarity of meaning.

Each interview was digitally recorded and fully transcribed verbatim. The data were read, reread and analysed separately by two researchers; one researcher (PC) using NVIVO software, and the other, a research assistant (PR), using a coding table. Both researchers used an iterative thematic approach [29] to identify and index common themes and categories. Each produced independent lists of codes and undertook constant and further refinement of coding. Any discrepancies in coding or interpretation of data between researchers were discussed and some minor modifications made until consensus was reached. Common themes (or uncommon themes) were checked against demographic and physical activity characteristics of the participants.

The use of two interviewers and two coders from different backgrounds was undertaken as this approach aimed at gaining a broader understanding of the phenomenon under study whilst limiting the potential risk of individual interviewers’ and coders’ epistemological perspective or professional identity impacting on the research [30,31].

## Results

32 podiatrists were mailed invitations and non-responders were followed up until 20 podiatrists accepted (62%). The participants interviewed (Table 1) included both sexes, were from all age ranges and had a variety of work practices. Pre-registration qualifications were attained across the Australian states as well as from the UK and New Zealand and ranged from certificate level to bachelor degree with post-graduate qualifications. A wide range of physical activity levels was reported with most participants claiming above recommended levels of more than 150 minutes per week.

**Table 1:** Demographic and physical activity characteristics

**Characteristics**	**n**	**%**
**Male**	8	40
**Age**		
< 25	2	10
25 – 35	6	30
36 – 45	4	20
46 – 55	6	30
>55	2	10
**Practice (full time equivalent)**		
< 0.8	5	25
> 0.8	15	75
**Practice type**		
Private only	16	80
Public only	2	10
Public/private	2	10
**Practice area (speciality)**		
General only	5	25
Mixed	15	75
**Qualifications attained in**		
Queensland	1	5
New South Wales	2	10
South Australia	4	20
Tasmania	1	5
Victoria	4	20
Western Australia	2	10
England	4	20
New Zealand	1	5
**Physical activity**	**mean**	**range**
*Activity type*	*Minutes per week*	
Vigorous	67	0 – 720
Moderate	433	0 – 2520
Walking	411	20 – 2520

Common themes extracted from the data were grouped under headings of: physical activity promotion role beliefs, physical assessment practice and beliefs, physical activity promotion practice, barriers and enablers, motivational factors, normative influences, effectiveness of promotion and knowledge, education and skills. Interview quotes have been selected as exemplars to represent each theme. Supplementary quotes may be sourced in Additional file 3: Table S2.

### Physical activity promotion role beliefs

Participants saw physical activity promotion as integral to their role as health professionals and to their professional role of keeping people moving through the management of foot conditions:

*“I think that we keep them on their feet. So that slogan, Podiatry – keeping people on their feet, is a good one, and I feel that if we can keep people moving as long as possible in their lifetime, they’ll remain healthier.”* (Pod 16)

The participants felt they needed to have a holistic approach to patient care as opposed to focusing on an isolated problem:

*“I think the fact that there is so much chronic disease around that we have to get better at making sure we see a person as a whole person and not just looking at their feet.*” (Pod 11)

They saw they had a role in giving patients information, advice and education on physical activity and its benefits as well as making suggestions or recommendations on physical activity options and encouraging patients to be physically active. They believed physical activity plays an important role in chronic disease prevention and management:

*“I think we’ve got a pretty big role, we see a lot of people who aren’t active and who have developed things like Type 2 Diabetes and heart problems, and problems with mobility.”* (Pod 20)

Additionally particular mention was made by the participants about the role of encouraging those patients with injury, disease or disability to continue with physical activity:

*“I think that generally we probably see populations that have come in with some type of injury or disease or disability, so it’s part of the role is to be able to educate them of ways that they can continue physical activity while being able to accommodate that disability or injury or whatever may potentially be reducing their current physical activity.”* (Pod 2)

Different beliefs about the limitations to the role were evident with some being unsure of their role and the boundaries surrounding their role. Seven of the participants did not consider physical activity assessment, exercise prescription or monitoring to be a role of the podiatrist, rather they were thought to be the role of other health professionals such as exercise physiologists, GPs and physiotherapists:

*“I guess I’ve tended to think that’s more for the physio or the GP, but I guess there is a place for us there. But it’s never something that I’ve really considered.”* (Pod 14)

Furthermore they saw part of their role was to refer patients on to other health professionals with more experience, particularly when the patient presented with high risk conditions:

*“…it depends on the person coming in really. If it’s someone who’s quite high risk, multiple complex issues, I think err on the safe side, and have to be a referral off to someone who is an expert in the area.”* (Pod 13)

### Physical activity assessment practice and beliefs

The participant’s decision to assess a patient’s physical activity level, was more likely made when the level of physical activity contributes to the presenting condition:

*“The problem they've got…will often inhibit their physical activity so that becomes part of the discussion about what they're doing and what they want to achieve in terms of where they want to end up being with the treatment.”* (Pod 12)

It may also depend upon the patient’s characteristics, such as medical history and age. For example, diabetic patients were more likely to be assessed:

*“Would probably be a middle age, over weight diabetic patient and recently diagnosed as well.”* (Pod 1)

Elderly patients and those that present with significant health issues or disability were less likely to be assessed:

*“Older people… if they’re coming in for a general treatment I’m not likely to assess their physical activity. I might encourage them to do more… whereas someone who’s coming in with a pain in their foot condition type of thing, I’m more likely to assess them.”* (Pod 14)

The way information about physical activity was gathered varied considerably. Assessment often involved informal conversation as well as practitioner-led questions:

*“I guess once they start talking to you and talk about their health problems, as most of them do, and I guess then you can sort of assess to sort of what level they’d be at and what they could do. That’s about it.”* (Pod 5)

Or more formally as history taking, particularly in the case of a diabetic or biomechanical assessment:

*“I guess you do that to a certain extent, probably not a huge written report, but when you see someone, particularly the biomechanics side of it, you are actually looking at what they do, and what they can do.”* (Pod 15)

Observation of the patient’s physical capabilities and movement patterns was also used as an assessment technique. Often the activity levels of the patient were inferred by appearance:

*“.... if you look at someone who’s coming in and they’re struggling to get into a normal chair, they’re obviously very bariatric, you’d be like, yeah I don’t think this person does much, it could be… but if you get someone who’s really trim and fit coming in wearing joggers, you tend to think, yeah potentially this person will go for a walk … You shouldn’t as a health professional, but they just… it’s just there, it’s just obvious.”* (Pod 13)

*“…but my ongoing geriatrics would be more like me gleaning information as they walk in, as they walk out, as they move from the chair to the other chair after we get their shoes and stuff on, so it’s me just watching everything happening.”* (Pod 16)

When physical activity was assessed formally it was part of an injury or biomechanical assessment and commonly the aim was to assess the duration, frequency, intensity and type of activity. It was less common to assess work-related activity, the level of sedentary behaviour or where activity was carried out.

Participants usually found it easy to raise discussion about physical activity levels and types with patients, as this was often relevant to presenting conditions.

Barriers to a useful assessment included lack of time and assessment skills along with difficulty in gauging actual levels and types of activity:

*“If people are retired and they don’t do much then sometimes it will be gardening and bits and pieces, that’s hard to figure out exactly how much activity they’re doing…”* (Pod 10)

There seemed to be a misunderstanding by the participants of what physical activity assessment entailed as some considered this to involve fitness testing for which they claimed a lack of skill.

Some expressed concern about the authenticity and genuineness of patients’ self-reported physical activity:

*“I think sometimes they say they’re doing a bit more than they probably are, but yeah, it’s human nature.”* (Pod 14)

### Physical activity promotion practice

Participants varied considerably in physical activity promotion behaviour however there was a noticeable unstructured and informal approach taken by the majority. There was also an overwhelming preference for advising walking as an activity along with swimming and cycling or the use of an exercise bike. Participants also reported tailoring their recommendations for individual patients with the advice given dependent upon the patient’s age, personal interests, current physical activity levels and capability, health conditions and injuries as well as potential risks to the patient:

*“I guess it’s just getting to know your clients and what they’re comfortable with and what you think they can handle.”* (Pod 10)

While three of the participants stressed the importance of all patients needing to receive the physical activity promotional message, the approach taken is often opportunistic. Many of the participants reported targeting particular patient types. For instance, it was typical for podiatrists to target those patients with diabetes, or other chronic diseases, who were overweight or who they assumed were sedentary.

Participants reported being less likely to promote to patients that they deemed either already active or unable to be active due to a serious health issue or where there was a potential health or safety risk to the patient:

*“You get someone with lots and lots of health problems that come in, like someone who’s got cancer, and they’re having treatment at the moment for cancer, they really don’t want to be fussed about knowing that they should do this and that for their diabetes. And I would not be bothered.”* (Pod 17)

Participants varied considerably in their follow up and monitoring of their patients’ activities. Follow up was generally approached opportunistically and informally during conversation with the patient when they came back for a return visit. Systematic follow up did generally occur as a part of an annual diabetic assessment or management of an injury or biomechanical condition:

*“I think there is a follow up for those with chronic disease in that you’re probably seeing them on an annual basis for their assessments. In terms of the more active group, from people coming in with injuries is definitely follow up because you’d follow them through probably the course of their injury, or at least a reasonable portion of it. But beyond that, probably not, they’re probably left to their own devices.”* (Pod 2)

Participants documented little in the way of their physical activity promotion other than specific recommendations related to the presenting condition. When asked this question, a few participants mentioned that they had not considered it a task they should be doing, however, they could see the value in doing so, particularly in follow up of patients. When it was documented, then it was within the patient notes or as part of a report to the patient’s general practitioner or management plan.

### Physical activity promotion barriers and enablers

Participants perceived the barriers to promotion, on their part, were associated with a lack of time, resources and knowledge of activity options:

*“Knowing what resources are out there and keeping them up to date as well. There are new things that come along that I don’t know about, activity groups and things like that. It changes if I’m working in a different setting.”* (Pod 11)

Also a lack of specific skills, especially exercise prescription knowledge and behaviour change skills:

*“Unless you’ve specifically trained in a particular area and have the skills and knowledge and expertise to be able to assist patients more in that field… but for many podiatrists they probably haven’t had that degree of undergraduate or possibly even postgraduate training. I think if they’ve got skills, knowledge and confidence in that area to be able to do it well then go for it, I think it would be great.”* (Pod 9)

Additionally there was a concern about the potential risks to patients and possible litigation brought about by the information given:

*“But I guess because there’s always the fear of litigation and saying, you know I think the recommended… putting a time, and putting a number on things, and without having the evidence background, the evidence base…and people might misconstrue the message that you’re trying to say.”* (Pod 13)

Perceptions of barriers presented by the patient were where the patient was perceived by the podiatrist as being unmotivated, unreceptive or having a negative attitude towards physical activity:

*“I think usually you can tell fairly early on, like within the first five or ten minutes whether someone’s going to actually listen to advice you’re giving them, or whether they’re just pretty negative and set in their ways and they’re not going to change no matter what you say.”* (Pod 20)

The participants varied in their perceptions of what did and would enable them to promote activity to their patients. Generally participants considered that the routine consult is an ideal opportunity to promote physical activity:

*“I think we’re in a really good position where we have the patient there in most cases for probably 15 to 20 minutes, and where we can chat to them while we’re looking after their feet, and we can suggest different forms of activity that we think might help them.”* (Pod 13)

Regularity of these consultations helps to build rapport and familiarity with the patient and provides ongoing opportunities to target and tailor the message as well as to follow up and monitor their promotional efforts:

*“We’re in an ideal position to be able to monitor them if they are active or becoming active because we see most people on a regular basis, whether it be every 12 months or every two months… we ask people regularly over a long period of time, so you do get a relationship with your patient.”* (Pod 8)

It was believed by some participants that they have better opportunities than other health professionals to promote activity due to regular visits and time spent with patients:

*“....musculoskeletal injury that the physios will see that with that fixed they’re discharged.... Whereas we keep seeing them every six, eight weeks, whatever, for the nail care. So we generally don’t discharge patients.....I think because GPs are so busy, we spend a lot more time with each individual person, that we have the ability to just reinforce those guidelines.”* (Pod 1)

Many participants recognised that the annual diabetic assessment was a good opportunity to promote physical activity. The patient’s level of motivation was often cited as a facilitating factor along with patient rapport. Others felt that having resources such as handouts and visual cues made it easier to raise and communicate the message. Having access to resources and knowledge of local activity options was also believed to make it easier to promote physical activity. A number of participants reported that they felt that formalised strategies along with training in physical activity promotion methods would improve promotional practice behaviour and efficacy:

“*Having some good strategies in place that you know work would make a difference, it would motivate you to do it more if you knew something had an 80% success rate and then you would do it.”* (Pod 12)

A few, particularly public practising participants, felt that a multidisciplinary team approach was beneficial:

*“It’s something that’s part of - certainly in Public Health - part of our ongoing management of these people. We work together quite closely with people like Diabetes Educators and the Endocrinologists and other specialists. We’re all pretty much on a similar page with the messages that we try to get out.”* (Pod 9)

### Physical activity promotion motivational factors

The more common reasons for podiatrists’ promotion were a desire to improve patients’ health through physical activity as well as personal and job satisfaction and a sense of achievement:

*“.. it makes me feel good to know that I’m helping, and this is why I studied Podiatry in the first place, to help people have a good quality of life. And people who can change their lives around will come back and they will generally tell you they’re feeling so much better and they can do more, and it makes me feel good. It makes me… it justifies why I choose to do this profession. That’s all I’m looking for, for my career.”* (Pod 2)

### Physical activity promotion normative influences

Participants reported varied sources of normative influence towards promoting physical activity although a number perceived their influence was gained through professional development events, general knowledge and from colleagues:

*“Well of course, even just going to conferences and hearing people talk about the importance of physical activity and making changes in the community, of course that provides a level of motivation to… for us to promote physical activity.”* (Pod 2)

### Effectiveness of promotion

The participants reported mainly gauging the success of their promotional efforts through conversational feedback and observational methods. Predominantly it included seeing changes from visit to visit, improvement in the presenting condition, improvements in chronic conditions and weight loss. Some of the more innovative observations mentioned of measuring effectiveness included the ability of a patient to be able to trim their own nails, the state of patients’ shoe wear and an increase in the callus build-up on the patients’ feet:

*“I was trimming their nails, because they were just presenting for that, and the diabetes is out of control, to losing a lot of weight and then being able to trim their own nails and been taken off insulin for diabetes…”* (Pod 1)

The participants exhibited a range of beliefs towards their promotional effectiveness from the negative:

*“To be honest, most people probably don’t change that much at all. Most people are probably either the same every year, unless someone was on a health kick one year and the next year they’re not or vice versa.”* (Pod 12),

To the positive:

*“I had a patient in last week who, on my advice, has been walking 20 to 30 minutes every day, has lost weight, he’s medication reduced, he’s really quite happy that I’ve got him motivated to go and walk every day, so.”* (Pod 20)

Three of the participants found it difficult to measure effectiveness:

*“I don’t know that we do it effectively, we’ll talk to them but how many people will then be motivated by that advice to go away and change their routine and habits? It’s a hard one to measure.”* (Pod 12)

### Physical activity promotion knowledge, education and skills

It was evident from the reports that there seems to be limited pre- or post-registration physical activity promotion education for podiatrists:

*“Unless you’ve specifically trained in a particular area and have the skills and knowledge and expertise to be able to assist patients more in that field… but for many podiatrists they probably haven’t had that degree of undergraduate or possibly even postgraduate training. I think if they’ve got skills, knowledge and confidence in that area to be able to do it well then go for it, I think it would be great.”* (Pod 9)

Even so, participants seemed to have a broad knowledge of the numerous physical and mental benefits of physical activity although many had a limited understanding of the specifics of the benefits and the current recommended guidelines. Participants felt they lacked skills and in particular they wished they had more training within physical activity assessment, exercise prescription, behavioral change, counseling and motivational interviewing:

*“…theoretically if I was going to go down the pathway of really doing proper physical assessments, I’d probably want to do a bit more continued ed, just to learn a little bit more, feel a bit more confident I guess.”* (Pod 15)

### Comparisons of themes and demographic data

The only obvious difference between themes across the different demographic attributes were between podiatrists working in the public vs private sector. Public sector practising participants made more mention of documentation of physical activity promotion:

*“Usually that’s in our management plans so any of our care plans we put together for our patients, in particular for Public Health… in private practice that’s just part of the medical records that you put together as part of their ongoing history and usually that’s on the front page and gets updated from time to time.”* (Pod 9)

Public sector practising participants also more often reported the influence for promotion coming from other health professionals and a team approach:

*“Those team roles and relationships that we’ve had and built up for a long time certainly benefit patients in many ways and benefit us in those inter-professional relationships. I think we all end up picking up other messages that have been passed on also so that team approach, I think, is a really good, positive thing for everyone around.”* (Pod 9)

The analysis of the data revealed no other obvious differences or similarities between common or uncommon themes and the demographic attributes.

## Discussion

To our knowledge this is the first study to provide insight into current practice of podiatrists with regard to physical activity promotion and the factors that influence and prevent podiatrists in enacting their physical activity promotion role. The findings demonstrate that the podiatrist’s unique patient-practitioner relationship appears to provide a significant opportunity for the provision of physical activity assessment, promotion and monitoring during regular routine clinical care. Podiatrists are receptive to their role in physical activity promotion and the profession is well placed, given an appropriate level of training, guidance and support, to play an important role in positively impacting the health behaviours of their patients. Physical activity promotion appears feasible in podiatry practice in terms of opportunity and acceptability to practitioners, but there is scope for improvement. Strategies to improve promotion need to consider the major issues, barriers and opportunities as well as provide a more structured approach to physical activity promotion by podiatrists.

The majority of participants had favorable attitudes towards their role in physical activity promotion and this was echoed by many showing interest in improving their knowledge, skills and practice in the area. A positive and supportive attitude of health professionals has been claimed to be an instrumental factor in promotional behaviour [32,33]. This finding is probably not surprising given that there appears to be a natural synergy between physical activity assessment and promotion and the podiatric role, as maintaining or improving mobility and enhancing the independence of individuals is considered core to podiatry practice. It is therefore interesting that seven of the participants considered that the role of podiatrists in physical activity assessment and promotion was limited. It was also revealing that the practice of physical activity assessment and promotion was not universal amongst podiatrists and lacked any real structure. This may be a consequence of there being limited information available to alert them to the role nor is there much in the way of any educational opportunities to give them the knowledge and skills to perform this role. In addition there are no physical activity promotion guidelines or policies for podiatry and furthermore there is a distinct lack of clarity around the role of all health professionals, in relation to the promotion of physical activity and related health behaviour.

The enablers of physical activity promotion specific to podiatry come from the unique podiatric interaction and relationship with patients. Problem nails, corns, callus and toe deformities are conditions that commonly require regular routine core podiatry care [34], the performance of which appears to provide an opportunity to counsel patients on their physical activity behaviours. It was interesting whilst some participants felt time was a barrier others were of the view that there was time during routine consultations to enable them to counsel patients. This highlights the need for further assessment of the feasibility and capacity of the delivery of promotional activities during consultations. Many of the presenting conditions often require regular six to eight week consultations over a lifetime which not only builds rapport with the patient but also offers the chance for ongoing physical activity counseling that could be targeted, tailored and combined with continued support. This approach has been shown to be effective in increasing physical activity levels, particularly in the short term [35,36].

Many factors identified as potentially influencing physical activity assessment and promotion in podiatry, are similarly identified in studies of other health professionals. In particular, the targeting of particular patients and taking an opportunistic approach to assessment and promotional efforts has been noted in many studies [9,10,32,37]. Studies have also shown that patients with particular characteristics, notably those who are overweight and those with chronic conditions, are more likely to receive physical activity counseling (13,28). This contrasts with recommendations that physical activity promotion be provided to all patients routinely by health professionals [38]. The description of current practice suggests a lost opportunity for podiatrists to potentially contribute to public health efforts to reduce the burden of chronic diseases by assessing, promoting or following-up physical activity with all patients rather than simply “as required” as in the case of the annual diabetic assessment or when it is only relevant to the presenting condition. Our data suggests that the reasons for this are diverse, ranging from podiatrist beliefs about their role and their effectiveness at physical activity promotion, to a lack of skills and educational opportunities.

The suggestion in the data that public sector podiatrists are more likely to document or be influenced by other health professionals’ promotion should be considered carefully. It is possible that public sector podiatrists in Australia do have more stringent documentation policies and procedures, and do collaborate with a more diverse spread of health professionals as compared with private practicing podiatrists. This observation may be useful in the future studies.

Once a patient has been targeted for physical activity promotion, the tailoring of advice towards patients’ characteristics and preferences reported by participants in this study has also been shown to be a common occurrence amongst primary care physicians [37]. However, the practice of tailoring advice as opposed to adhering to a standard message has been associated with increases in physical activity levels in the short-term [35], so such tailoring may be desirable. The tailoring of advice to the patient has also been advocated in diabetic education [39,40] and foot health education for patients with rheumatoid arthritis [41].

Barriers to physical activity promotion perceived by the participants are common amongst other health professionals including the lack of time, knowledge and skills, resources and perceived lack of effectiveness of their efforts and limited patient receptiveness [8,10,11,42]. These inhibiting factors could potentially be addressed though a number of measures including:

1. Training that improves skills, knowledge, effectiveness and consequently confidence.

2. Improved access to resources including written education material and knowledge of local activity options.

3. Development of a systematic approach to podiatric physical activity assessment.

4. An evidence-based formalised strategy for physical activity promotion that is designed to give guidance to podiatrists whilst maximising the effectiveness and efficiency of their promotional efforts.

In support of this, studies with primary care physicians have shown that interventions that include written materials for patients, considered behaviour change strategies, and provide training and materials, have been shown to be effective at increasing levels of physical activity [43].

It is important that consideration be given to the issues of clinical governance [44] of physical activity promotion practices in light of comments made by the participants regarding giving physical activity advice, prescribing exercise, exercise counseling and the lack of education and training. If physical activity promotion by podiatrists is to be encouraged, then podiatry organisations may need to provide podiatrists with appropriate educational and training opportunities to ensure that physical activity promotion is performed safely and in an evidence-based way. As with any other aspect of their professional practice, podiatrists themselves also need to ensure that they have sufficient knowledge and skills in this area and are aware of the potential risks of and responsibilities associated with physical activity promotion.

While this study is limited to one Australian state, we included participants with a diverse range of demographics and of different physical activity levels from various areas of the state and our findings were consistent with previous research, making us confident that our results are broadly generalisable to Australian podiatrists. As with other physical activity promotion studies involving self-reports [13,45] there is the possibility of social desirability bias, however we believe that this may not be an issue with this study as there was a wide range of reported levels of promotional behaviour and no podiatrist reported high levels of promotion.

## Conclusions

The podiatrist’s unique patient-practitioner relationship appears to provide a significant opportunity for the provision of physical activity assessment, promotion and monitoring during regular routine clinical care. Participants were receptive to their role in physical activity promotion and the profession is well placed, given the appropriate level of training, guidance and support, to play an important role in positively impacting the health behaviours of their patients. However, while physical activity promotion appears feasible in podiatry practice there is scope for improvement in promotion behaviour. Strategies to improve promotion need to consider the major issues, barriers and opportunities as well as provide a more structured approach to physical activity promotion by podiatrists. In addition more work needs to be done to ascertain the actual capacity and feasibility of podiatrists being able to carry out physical activity assessment, promotion and monitoring as part of their clinical role as well as to measure the efficacy and impact of their promotional efforts with patients.

## Competing interests

The authors declare that they have no competing interests.

## Authors’ contributions

PC, VC, TW participated in the conception and design of the study. PC performed acquisition of data and statistical analysis. All authors helped draft and approved the final manuscript. PC was the principal investigator. All authors read and approved the final manuscript.

## Supplementary Material

Additional file 1: Table S1 Interview schedule. Click here for file

Additional file 2 Participant demographic and physical activity characteristics survey. Click here for file

Additional file 3: Table S2 Supplementary quotes. Click here for file
