# Supplementary material for: Understanding the physical activity promotion behaviours of podiatrists: a qualitative study
Source: J Foot Ankle Res. 2013 Sep 9;6:37. doi: 10.1186/1757-1146-6-37 (PMC3846794; doi:10.1186/1757-1146-6-37)
Supplement: Additional file 1: Table S1 — Interview schedule. [file 1757-1146-6-37-S1.docx]

| **Additional file 1: Interview schedule** |
| --- |
| **Notes: Key questions are indicated in bold and possible prompts in oblique. The wording of the questions and the use of prompts is expected to vary from interview to interview depending on information forthcoming from the participant** |
| 1. **What comes to mind when I mention physical activity promotion?** |
| 1. **What role, if any, do you think podiatrists’ have in physical activity promotion?**   *If no role, why do you feel that? Both no role and yes a role prompts: Do you think podiatrists’ have a role in; giving advice, assessing physical activity, giving recommendations, monitoring or following up on recommendations?* |
| 1. **Can you tell me about how you might assess your patient’s physical activity level?**   *If no, Can you explain your reasons? If Yes; How do you decide whether or not to assess a patient’s physical activity levels? Are there any groups that you are more or less likely to assess physical activity? How do you raise the issue with your patients? How would you determine a patient’s physical activity level? What types of questions do you use? What things do you aim to establish?* |
| 1. **Do you promote physical activity to your patient?**   *If no, tell me why you don’t? If yes, Tell me more / give me an example / In what situations do you promote physical activity? When do you choose not to promote physical activity?* |
| 1. **What physical activity recommendations do you make, if any?**   *If none, is any particular reason why you don’t. If Yes; How do you follow up your recommendations? Do you involve others in your practice in physical activity promotion, like colleagues or staff?* |
| **6)** **How would you document your physical activity promotion or patient’s physical activity level or physical activity recommendations?** |
| 1. **What other types of health promotion do you use in your clinical practice?**   *For example; nutrition, weight control, smoking, stress, drug and alcohol* |
| 1. **What do you think are the benefits of physical activity?**   *Do you think physical activity has a role in the prevention and management of chronic disease? .... if so, what role? How much physical activity do you think people should be doing?* |
| **9)** **Are there advantages to promoting physical activity to patients?**  *What are the advantages to your patient / to you in promoting physical activity to your patients?* |
| 1. **Are there disadvantages to promoting physical activity to patients?**   *What are the disadvantages to your patient/ to you in promoting physical activity to your patients?* |
| **11) Do you think physical activity promotion works better with some patients as compared to others?** |
| 1. **Are there any *others* that would influence you in the promotion of physical activity to your patients?**   *(Are there any other groups? Other people? - Examples of others; your colleagues / professional association / other health professionals / GPs / family / friends)* |
| 1. **What role do you feel these *other*s think podiatrists should have in physical activity** **promotion?**   *Role in; giving advice, assessing physical activity, giving recommendations, referring, monitoring or following up on recommendations?* |
| **14) What makes / would make it easier for you to promote physical activity to your patients?** |
| **15) What makes / would make it harder for you to promote physical activity to your patients?** |
| **16) *Could you give me an idea about h*ow effective have you been / do you think you would be in increasing physical activity in your patients?**  *Tell me more / Why or why not?* |
| **17) Tell me about what if any physical activity promotion resources do you use?**  *If so, tell me about what you have/have access to?* |
| **18) Where would you go / or have you gone to find information or education about physical activity or physical activity promotion?** |
| **19) Before we finish today is there anything else further you would like to tell me about any of the things we’ve talked about today or anything we haven’t discussed that you feel is important?** |
