# Supplementary material for: Understanding the physical activity promotion behaviours of podiatrists: a qualitative study
Source: J Foot Ankle Res. 2013 Sep 9;6:37. doi: 10.1186/1757-1146-6-37 (PMC3846794; doi:10.1186/1757-1146-6-37)
Supplement: Additional file 2 — Participant demographic and physical activity characteristics survey. [file 1757-1146-6-37-S2.doc]

**BRIEF** **SURVEY**

Please take a moment to fill out a few personal and practice details prior to your interview. This survey will be collected by the interviewer at the time of your interview.

***Personal Details***

**Sex: M / F**

**Tick a box as per your age: < 25** ☐  **25–35** ☐  **35–44** ☐ **45–54** ☐ **> 54** ☐

**Tertiary Qualifications _____________________________________________________**

**Educational institution/s _____________________________________________________**

***Practice Details***

**1) In what kind of practice do you work? (please tick as many as are applicable)**

**Private practice** ☐ **Community based** ☐ **Public hospital** ☐ **Other** ____________

**2) What is your area of practice? (please tick as many as are applicable)**

**General** ☐ **High risk** ☐ **Biomechanics** ☐ **Surgery** ☐ **Other** _____________

1. **Approximate practice days per week ________**
2. **Number of years in practice _______**

***Your Physical Activity***

The next questions are about the time you spent being physically active in the last 7 days.

They include questions about activities you do at work, as part of your house and yard work, to get from place to place, and in your spare time for recreation, exercise or sport.

In answering the following questions:

“**vigorous:** physical activities refer to activities that take hard physical effort and make you breathe much harder that normal.

“**moderate”** activities refer to activities that take moderate physical effort and make you breathe somewhat harder that normal.

1a. During the last 7 days, on how many days did you do **vigorous** physical activities like

heavy lifting, digging, aerobics, or fast bicycling,?

Think about *only* those physical activities that you did for at least 10 minutes at a time.

**________ days per week**

1b. How much time in total did you usually spend on one of those days doing vigorous physical activities?

**_____ hours ______ minutes**

2a. Again, think *only* about those physical activities that you did for at least 10 minutes at a

time. During the last 7 days, on how many days did you do **moderate** physical activities

like carrying light loads, bicycling at a regular pace, or doubles tennis? Do not include

walking.

**________ days per week**

2b. How much time in total did you usually spend on one of those days doing moderate physical activities*?*

**_____ hours ______ minutes**

3a. During the last 7 days, on how many days did you **walk** for at least 10 minutes at a

time? This includes walking at work and at home, walking to travel from place to place,

and any other walking that you did solely for recreation, sport, exercise or leisure.

**________ days per week**

3b. How much time in total did you usually spend walking on one of those days*?*

**_____ hours ______ minutes**

The last question is about the time you spent sitting on weekdays while at work, at

home, while doing course work and during leisure time. This includes time spent

sitting at a desk, visiting friends, reading traveling on a bus or sitting or lying down to watch television.

4. During the last 7 days, how much time in total did you usually spend *sitting* on a

**week day?**

**____ hours ______ minutes**

***Thank you***

***Please keep this survey for collection at the time of your interview***
