# Supplementary material for: Understanding the physical activity promotion behaviours of podiatrists: a qualitative study
Source: J Foot Ankle Res. 2013 Sep 9;6:37. doi: 10.1186/1757-1146-6-37 (PMC3846794; doi:10.1186/1757-1146-6-37)
Supplement: Additional file 3: Table S2 — Supplementary quotes. [file 1757-1146-6-37-S3.doc]

| Additional file 3: Supplementary quotes – (Pod = Podiatrist) | | |
| --- | --- | --- |
| **Physical Activity Promotion** Role Beliefs | | |
| Role as a health professional | | “…. being a health professional rather than just a podiatrist that looks at someone's feet ….it's definitely something where every person that the person sees, every health professional, that message should be coming across and we should be doing as much as we can.” (Pod 11) |
| Holistic approach vs focusing on an isolated problem | | “We could actually include that in our more clinical side of things rather than just our conversation side of things so we can give them the more holistic approach if we know a lot more of what they're involved in and then we can help them on a lot of different levels. I think a lot of clients come to us for one thing, not realising we can help with something else.” (Pod10) |
| Role in giving information, advice, education, recommendations and encouraging | | “It's not to say I'm about to set up a physical activity program for anyone but sometimes you might need to help plant the seed to look at, to help someone facilitate starting off a new program or maybe even thinking about whether or not they could do exercise in a pool, not necessarily swimming.” (Pod9) I they’re doing  “I don’t sort of set goals for them or anything like that, but I say, “It would be good if you could have a walk around the block,” or something like that. Or if they say that they’ve... they park at the car park and they walked here, well I’ll say, “That’s good, you know that’s all good exercise.” Yeah, so it’s more reinforcing, you know helping what they’re doing, and giving them a bit of encouragement I think.” (Pod 14) |
| Role in chronic disease prevention and management | | “Certainly with diabetes as the epidemic that we have we, as podiatrists, have a huge role in offering preventative sort of treatments …. a lot of it is talking about physical activity and I would think that any of my colleagues would think that would be our role, to push for physical activity as a form of treatment.” (Pod 7) |
| Role limitations | |  |
| Physical activity assessment | | “I haven’t ever thought about that actually. I think that would be more a role for a Physio or an Exercise Physiologist.” (Pod 20) |
| Exercise prescription | | “…general Case Managers, so probably your General Practitioners, your Medical Specialists, maybe other Allied Health Professions are involved in physical activity prescription, like Physiotherapists probably would feel that maybe it’s not so much the Podiatrist’s role to specifically advise on physical activity specifically, or to specifically advise on physical activity.” (Pod 2)  “I think that would be more a role for a Physio or an Exercise Physiologist.” (Pod12) |
| Monitoring | | *“I would love to be able to monitor people, you know, get them to mark down how much they’ve done and give them a tick at the end of the time and do that sort of encouragement, but I don’t know that that’s our role.” (Pod7)*  *“I would assume that the consensus would be that there’s a role in promoting physical activity. I think that less people would probably feel that Podiatrists have a role in prescribing physical activity, which is probably fair enough. And depending on the... and probably not so much in the follow up, I think it depends on the, I guess it depends as a general sort of follow up and monitoring of physical activity and levels I think that would be expected from others. But in terms of specifically reviewing and modifying exercise programs, I think that’s probably not something that’s assumed that Podiatrists would do.” (Pod 2)* |
| Physical activity assessment practice and beliefs | | |
| Decision to assess | | |
| When related to the presenting condition or affecting or affected by mobility | | “...if their activity levels are affecting their foot health like if they're walking when they shouldn't be walking then I think we have a role in assessing that level of activity as well.” (Pod 11) |
| Dependent upon patient type | | “Probably different things in different populations, so probably more in your more active population that come in with say a sporting injury, it would be to look at training loads or exercise loads, how it’s distributed between different types of activities. So whether their injury may be as a result of too much of one particular type of activity. Then maybe in groups where you might be doing, say for example a diabetes assessment of neurovascular function, might be more around generally how much aerobic exercise they’re doing. And then maybe in a group which maybe people have neuropathy or who are at risk... their feet are at risk, it might be more specifically looking at the type of activity that they’re doing, so weight bearing activity might be more dangerous for them than others, so... and looking at whether they’re doing a sufficient amount of safe activity...” (Pod2) |
| During a diabetic/ neurovascular assessment | | “Depends on what they're seeing me for. If they're seeing me for an injury or a diabetes assessment then that's one of our standard questions that we ask about what sort of activity levels they have….for diabetes assessment we have a form and it should be asked every time we do one of those.” (Pod11)  “So it’s something that I talk to all my patients with diabetes about, how much activity they’re doing on a daily basis.” (Pod20)“And then if they come in, if they’re a diabetic and coming in for a yearly check-up, well then you can check on them again.” (Pod17) |
| **Less likely to assess** | | |
| The elderly | | “I think the elderly is... or the aged, say those over 65 are probably a group that you assume are less active and sometimes you may not cover an accurate or detailed activity as you would with maybe more active populations that come through.” (Pod2) |
| When there are significant health issues and or disabilities | | “…maybe a little bit more complicated for us to get somebody [assessed] who is very much maybe chronically in pain that would be a quite hard. It would mean that it’s not harder for your thinking and your assessment; it’s just what the key priority would be at that point. And sometimes, unless you do approach somebody with that, you’ve obviously got to be very careful in the way that, you know the jargon and the language that you use.” (Pod4) |
| Assessment process | | |
| Informal | | *“How I would I assess it? Just by discussion. I mean, really you can look at people…” (Pod7)* |
| As a part of history taking | | “Well we have in our forms that we fill in, you know for us it’s... our safety net’s always the form, so look it’s our obligation to fill in this form, this is a new patient assessment.” (Pod13) |
| By observation (inferred by appearance) | | “I think that's because they probably appear more frail and so it's not to say they shouldn't be, they're probably just not because they appear as though they are less active and so you make a judgement on what you think they're capable of doing. They might be capable of doing a lot more than you actually take them at face value as being able to do.” (Pod12) |
| Facilitators | | |
| Easy topic to raise with patients | | “It’s something that comes up easily, it’s just, you know…do you walk? Because people use their feet, physical activity comes up very easily, it’s not something that is hard to raise I don’t think.” (Pod7) |
| **Barriers** | | |
| Lack of time | | “…depending on the actual person, the six minute walk test is... gives a pretty good indication of physical fitness for the more immobile elderly people. So depending on how far they can walk in the six minutes. This is actually not something that I’ve done for a long time because frankly it’s something that I’m finding I don’t have the time.” (Pod1)  “I really don’t have time in my practice to set up special little sessions to assess mobility.” (Pod16) |
| Lack of training and skills particularly in assessing physical fitness | | “I just don’t think I’ve got the training or the knowledge to be assessing that accurately… I don’t think would ever have done that. If you specifically said, alright what clients have you assessed per their capacity? Have you measured walking distance – sit up to stand exercises or beep testing type things to measure their capacity, I’ve never done it as a podiatrist. I think, what I suppose I have done is looked at people and thought about, and discussed with them what activities they do, do and what their trying to achieve and if there’s limitations on their ability to do that working out why that is.” (Pod6)  “Well that’s where it comes to a bit of a sticky point because with little training in assessing people’s physical fitness.” (Pod1) |
| Difficult to assess physical activity | | “Sometimes… if people are retired and they don't do much then sometimes it will be gardening and bits and pieces, that's hard to figure out exactly how much activity they're doing…we try and tease that out as well if you think the activity is…” (Pod10) |
| Concern about the authenticity and genuineness of patients’ self-reported physical activity level | | “…the only way that I would know how to do that, it is just to ask them in detail, their responses really to how much their actually doing and expecting them to tell me more than is actually accurate.” (Pod19)  “Sometimes it's tricky, you're trying to get people… what they say that they do as opposed to what they actually do. I start off by asking people what they do, if they go for a walk and how long they walk for or if they do any other sort of exercise.” (Pod11)  “I’m amazed how people think they do a lot and they actually don't do much at all…I guess you also make a judgement call on how honest they are, you have to assume the person is being honest with you but on some of the assessments we'll do gait assessments and things like that and if people are having difficulties with that you then have to question whether they're doing the level of activity that they state that they're doing. Usually it's off that initial assessment, it's a subjective assessment where we go through and ask them a series of questions based on that. That works pretty well, most people are not going to lie because they're there for their own benefit.” (Pod12) |
| Physical activity promotion practice | | |
| Recommendations tailored for individual patients dependent on: | | |
| Age | “…if we’re dealing with geriatrics, we do encourage them to walk, to move around for themselves, and we create the environment that would encourage them to do that as comfortably and regularly as possible.” (Pod16)  “…it doesn't necessarily mean being able to run 50km but for some patients - depending on their age and stage - it could be the difference between being able to do basic general day activities because they have the strength and mobility and can stand up on two feet without falling over. It can mean different things to different people at different ages and different stages.” (Pod9) | |
| Interests | “…helping them fulfil also what gives them joy and fulfilment in life is also a part of what is physical activity and movement .”(Pod16)  “…matching up activities that they may have an interest in or sometimes planting the seed about joining a group of walkers, for example, or finding a buddy to help with going out and walking the dog on a regular basis. Finding something they enjoy is probably the biggest thing. It is very much an individual thing but, again, it's how you deliver the message. Is it appropriate to that patient? Have you taken the time to find out about them and what their interests are? This comes from a bit of public health background, I guess, and work around chronic disease management. It certainly is about how you do deliver that message to that person and finding out what interests them, what makes them tick, what's going to light their button to go "Oh, yeah, activity.” (Pod9) | |
| Current physical activity levels | “So working out with people what they're doing currently and then trying to goal set with them about stuff they could be doing a bit more of or work out when they can increase activity. (Pod11) | |
| Physical capabilities | “It is, to talk about what they can't do and maybe what they should be doing as well. Sometimes people’s expectations of what they want to do and what they think they should be doing is actually less than what they should be doing.” (Pod12) | |
| Health conditions and injuries | “And quite often a lot of people that have musculoskeletal injuries to their foot, I write an exercise program for swimming or non-impacting activities, like exercise bike, or cycling and so on. Some of that, if they’re brand new to it, haven’t really done exercise, so I tend to ….suggest exercise that can be done. (Pod1)  “…but then modifying that for people who have maybe specific health conditions that would affect their ability to do those activities, or maybe being able to provide advice on activities that would be most suitable for their health conditions.” (Pod2)  “I mean obviously if you’ve got a 60-year old female, overweight, diabetic, you don’t want to suddenly say to them, ‘OK, you’ve got to go out jogging five times a week’ ‘cause it’s not going to happen. But if you say, ‘well if you could make half an hours walk five times a week’. So, I tend to think its more physical activity is kind of tailored to the individual rather than broad-based.” (Pod8) | |
| Potential health and safety risks | “I mean, obviously if they’re at a high risk of falling or something like that you might, yeah, suggest maybe that they do a bit of hydrotherapy rather than anything else or something like that.” (Pod8) | |
| Where promotion to patients is more likely | | |
| All patients | “I don’t think there’s necessarily, people that I wouldn’t, because I think most people would benefit from some form of physical activity.” (Pod8)  “To be honest I don’t think there are any groups that would be more or less likely for you to approach.” (Pod4) | |
| Diabetic patients | “I do actually really promote the daily activity with patients with diabetes, especially new patients that come in. I think that’s really important.” (Pod20) | |
| Chronic disease | “…circulation problems perhaps, something like that, have a bit more exercise and get the blood flowing a bit more. (Pod14) | |
| Overweight patients | “…a patient says to me, “I need to lose some weight,” then I would say just encourage them to be more physically active.” (Pod14) | |
| Sedentary | “One’s who are sedentary and, you know, depending on the patient of course, whether or not they’re debilitated by a condition and they pretty much can’t walk anyway.” (Pod3) | |
| Where promotion is less likely | | |
| Patient already active | “I think when you see a lot of kids or teenagers you probably don't have to promote it as hard because the majority of them… 99% of the teenagers you see are pretty active sorts of teenagers and that's less an area where we have to promote it, as such.” (Pod10) | |
| Patients with serious health issues (chronically ill, chronic pain) | “Well, certainly not someone who was in an acute phase of rheumatoid arthritis or something like that, and obviously they would say that they wouldn’t be able to do anything in that situation. But I would say, “Well when things settle down, perhaps try and go for a walk or something like that.” (Pod14)  “I think in severe cases of rheumatoid arthritis. For example, I have a patient right now who’s constantly got inflamed bursas beneath her metatarsal heads, with chronic necrosis and sloughing and exudates, I would say she should rest, be non weight bearing at times like that. So depending on symptoms, difficulties with healing of lesions on the plantar aspect of the foot may require an adaptation. If we can’t get weight deflection, they have to rest.” (Pod16)  “I guess people in chronic pain can be a bit negative about physical activity, and so perhaps it’s going to be harder to get them to do something than someone who’s feeling reasonably fit anyway.” (Pod14)  “I think patients who are having issues like they’ve got unstable blood pressure or their having cardiac problems, or they’ve had a stroke, I’m really reticent to promote physical activity if the GP isn’t involved as well.” (Pod20) | |
| Patients with a health and safety risk | “…but I think the limitation there is that there’s other factors such as often there might be heart conditions involved, respiratory conditions, falls risk…” (Pod2)  “…it depends on the person coming in really. If it’s someone who’s quite high risk, multiple complex issues, I think err on the safe side, and have to be a referral off to someone who is an expert in the area. If it’s someone relatively healthy, then I’d be like yep, just do it.” (Pod13) | |
| **Follow up practice and beliefs** | | |
| **Informal and opportunistic approach** | *“As far as follow up is concerned, I think that's part of just normal conversation in your consult time with patients where they're coming in for their primary problem or issue that you're managing and as part of that you'll be asking have they followed up with anything or how they're going with that program or what's been happening in their life and stuff. I think there's always that follow up and interest in people at personal level…” (Pod9)*  *“Probably not formally, no. So, again it depends on the nature of what they’ve actually come to see me with. If it’s just someone in general chit chat while I’m doing a routine consult I probably would tend to forget to follow that up unless I’ve made a specific note of it.” (Pod3)* | |
| **As part of a management plan** | *“I think our role covers being able to follow up on it, so to be able to provide the advice, potentially prescribe in some cases, and then to follow up as part of the management plan, or at least to ensure that it’s followed up by someone.” (Pod2)* | |
| **Documentation** | | |
| **Beneficial for follow up** | *“…you would recommend they come back in six to eight weeks and then you can also, like I always make really extensive notes, so to write down exactly the patient said, what you found, what actually happened, what you plan to do and then it will be easier for you to follow up whether there’s been any improvements or whether they think that things are staying the same.” (Pod4)* | |
| **Report to general practitioner** | *“I would just as a standard process on their medical records, I would be documenting that I’ve recommended or advised someone of a specific recommendations. I would often regularly write that to GPs, ‘cause I suppose GPs are often the gate keeper of a lot of elephant information and feel that if I’m including aspects of that persons care or physical activity that I think the GP should be made aware of that. And it’s also an opportunity to highlight that I just are considering those sorts of things outside of, you know, perhaps what a GP may think podiatrists do, so I use that as a promotional thing for podiatry as a profession I suppose, as well as me as a practitioner.” (Pod6)* | |
| **Barriers** | | |
| Time | “Well the same reason but to a lesser degree, that why the GPs aren’t doing it? It’s time constraint...” (Pod1)  “I’m always ten minutes behind for my next patient and you treat what’s in front of you and get them out the door and that I think tends to happen too much, in particular, the way I’ve developed my work. To give as best sort of exercise recommendation or prescription as I should in a less busy practise. I imagine I could do a lot more towards promoting exercise, and particularly in my circumstances you know of being commission based, you don’t get the patients through the door, you don’t get income, so... the pressures on a bit to keep the patient turnover going, so I guess my work structure is probably a bit of a barrier there.” (Pod1)  “…time factor with your appointments is a big thing. There are some clients you feel like you could sit here all day and talk to and encourage and do things with and then you'll suddenly go "Oh, I had them booked in for a 20 minutes appointment and we've been sitting here for 45. Far out." Sometimes you need to think… especially with new clients, if you don't know what the problem is then maybe booking them in for longer. If the person has an issue you'd really like to discuss but that wasn't what they came in for in the first place then getting them to come back or next time booking them in for longer so you can spend a lot more time with them.” (Pod10) | |
| **Lack of resources** | *“…it’s about me trying to source out where I’d get the information and think well that’s useful, but more often than not you would run out of those brochures and never use them again, so it’s not like I’ve got a permanent supply of information or brochures, or flyers or whatever available for people, so it’s just a timing thing, sometimes you have it, sometimes you don’t.” (Pod 6)* | |
| Lack of knowledge of activity options | “So there’s a lot of resources out there, but being aware of what they are and how well they’re promoted is another issues. I don’t just mean me promoting it to the client, but how well they promote them to practitioners and to a great degree, unless I go and source that information it’s not presented to us in any formal way, or there’s no specific resource where I can go, alright, I want to promote physical activity, what location do I go to get details around that, ‘cause it seems to be a pretty hotch-potch kind of, there’s no integrated location that I’ve found anyway, that I could say, alright, I can get information personally as a practitioner or I can advise my client and say why don’t you look at these areas to chase information. I suppose, for me more often than not I either stumble across them… I think time and also the reliability of what you’re offering somebody. Ensuring that the information you’re actually providing is individually appropriate and that you’ve considered a range of issues before, issues around safety and capacity and things like that before you actually…” (Pod6)  “If you haven't got the information and knowledge about what's around. I'm not an expert in planning someone's physical activity program but it's knowing who you can turn to and who you can go to for appropriate referral, that's probably the biggest barrier, I think. There are lots of things you find out by yourselves but there is probably lots of other stuff out there that's going on that you don't know about either.” (Pod9*)*  *“So you need to be informed yourself of what’s out there so that you can promote it….then that’s got to be updated all the time.” (Pod7)* | |
| Lack of skills and knowledge | “And I guess it’s the fear of not being trained in a physical activity, and not having a thorough history about patients, like other in depth about their cardio, their lung function, or respiratory function, and we’re talking about lots of high risk patients.” (Pod13)  “I guess it all comes back to things like behavioural change and it’s not an area which I’ve done any specific training, and it’s just been touched on in different elements of thing, but not... I don’t feel as though I have the skills in behavioural change...” (Pod2)  “I haven't got the skills or the knowledge to look at putting together some sort of exercise program.” (Pod9)  “Unless you've specifically trained in a particular area and have the skills and knowledge and expertise to be able to assist patients more in that field… but for many podiatrists they probably haven't had that degree of undergraduate or possibly even postgraduate training. I think if they've got skills, knowledge and confidence in that area to be able to do it well then go for it, I think it would be great.” (Pod9)  “I think time and also the reliability of what you’re offering somebody. Ensuring that the information you’re actually providing is individually appropriate and that you’ve considered a range of issues before, issues around safety and capacity and things like that before you actually…” (Pod6) | |
| Fear of litigation | “…these days with litigation and stuff, you know something happened and you told them to do something, and they came back and said, “Oh I’ve done this,” and I guess you’d have to watch.” (Pod15) | |
| Patient lacks motivation | “Well some people haven’t got the motivation, they’re lazy, they’re probably the two main factors, yeah, motivation and laziness and obviously you’ve got to make the time, you’ve really got to want to do it…” (Pod5)  *“…people that are proactive and positive and energetic and want to do everything they can to improve their health would I think... I think scenario would have a very good outcome. But the opposite to that personality or the low energy person, or the poorly motivated I’d probably expect to have a poor outcome.” (Pod19)* | |
| Patient is unreceptive | “Some clients don't want to hear it. A lot of clients are in denial and it's not exactly a disadvantage but if you over push something too much then your clients aren't necessarily going to want to return to you because they don't want to feel like they're getting a lecture every time they see you so you've got to tread carefully.” (Pod10)  “The client has to be receptive to it. I'm a strong believer that no one can help you until you're prepared to help yourself and so if the client is not prepared to listen you can bash your head against a brick wall all you like.” (Pod10)  “Like, you know, it’s much easier to take a tablet. You’re banging your head against the wall with some people, but I would still work on it.” (Pod7) | |
| Negative attitude to activity | “I think it’s difficult to be able to categories patients, but obviously you’d have some patients that maybe less willing to participate in physical activity than others for all sorts of reasons, and it’s more difficult to be able promote physical activity to that crowd, it doesn’t mean that you shouldn’t, but it’s more difficult to…” (Pod2)  “…some people just don't like activity. "I don't like going running, that's just how I am." Some people don't like going walking, that's just how they are.” (Pod11) | |
| Enablers | | |
| Opportunity during routine consults | “Well you’ve got them sitting in a chair for 20 minutes or whatever, half an hour, and you can talk about lots of things, but you can – not put them on the spot – but you can talk about things you want to.” (Pod15)  “So I think we’ve got to, because we’ve got a captive audience over half an hour.” (Pod20)  “I think as well as podiatry, yeah, people obviously do come for that particular problem, but very quickly  you start talking about other areas so, it is easier that way to rope that into it.” (Pod4)  “So we can say, ‘how have you been going?’ Just in the course of conversation and in the course of a consultation, it’s not having to put them on a spot it’s just part of the general conversation that you may have.” (Pod8) | |
| Regular consults | “Usually they come say every six to eight weeks and perhaps we could review that with them and see whether they were able in that period to increase, or whether they decreased, what problems they had to do that or not facilitate that. And obviously that generally might motivate them or keep them on the right path and we could advise them on what other things they could do.” (Pod4)  “And because of us reviewing for the nail care and so on, again and again, it’s more likely that we are going to see those patients again and again...” (Pod1)  “So it’s kind of like the patients that we see most of the time, and we see on a regular basis, and the ones we have had opportunity to build rapport, of the really, really high risk category, and most of the patients that might potentially have room for developing that kind of relationship and recommending.” (Pod13) | |
| Resources | “I think visual things are really good. I find, for example, I have sports shoes sitting over there and people see that and ask me about it all the time. They might not be interested in sport in particular, but they'll see the shoe and ask me why I have it there and if it's a good shoe and you use it as a visual thing to lead into conversation. Having things around that clients will ask about to stimulate conversation towards the direction you want to go, I think that helps a lot. Visual aids are good.” (Pod10)  “…so having the information .. whether it's some sort of brochure or a website - and bringing it all together would be the most helpful and the easiest way to be able to get information out.” (Pod9)  “I think a lot more pamphlets and handouts with literature about the benefits of exercise.” (Pod1) | |
| Implementation of formal strategies | “I think a structured, some kind of structured plan to fitness of starting, for example, with so much walking, perhaps some weight bearing exercises. I think just a program I suppose that I haven’t developed, that somebody else has developed, that has better knowledge in that area of what’s appropriate. And not just for one age group, through a number of age groups as well. I don’t feel I really have that, the knowledge to recommend that. So I think some guidance with that would help, that’s probably the main thing, that’s probably the main obstacle really.” (Pod20) | |
| Training | “I think I'd like to increase my skills in motivational interviewing so I would do some training in that, I think it's a good way to be able to ask people questions so they can… do that. I think chronic disease models are useful, they all have different ways of getting to the same ideas.” (Pod11)  “I think getting back to the behavioural change, that’s something that as Health Professions, well as a Podiatrist, we don’t really get... I don’t think we got any undergraduate training in it, and we learnt some basics of it …… well I feel as though I would benefit greatly from being able to do... having more skills and knowledge in that area, so whether that’s a component of undergraduate degrees that should be focused on more.” (Pod2) | |
| Multidisciplinary team approach | “I think if I actually worked in a multidisciplinary practice it would be a lot easier, with a Physio and an Exercise Physiologist and with a GP.” (Pod20) | |
| Influencing factors | | |
| Desire to improve patient’s health | “…probably more so with diabetics, because obviously it’s more important for them to keep their weight down and to do the exercise to get their blood glucose levels down and maybe reduce the need for medication.” (Pod5)  “I think if they're more physically active then it generally would mean they're less likely to be a complex case to manage if they're active and don't have as many co-morbidities which is probably less time consuming for a practitioner, less balls to juggle as far as management goes.” (Pod11)  “I guess the advantage we’d have patients who are much more proactive about their just general health, and we’d see I guess better outcomes long term with things like diabetes and heart disease.” (Pod20)  “Listen I think that that’s probably the group that would promote it more in because that tends to be the group that are less active and it’s going to be of more benefit to them generally in terms of all of their chronic health conditions...” (Pod6)  “You get that over time with individuals, so I guess you care about them and if you can improve their quality of life you do, basically.” (Pod8)  “I think if they're more physically active then it generally would mean they're less likely to be a complex case to manage if they're active and don't have as many co-morbidities which is probably less time consuming for a practitioner, less balls to juggle as far as management goes.” (Pod11)  “I think it’s really important for old people to, I guess just maintain their fitness and strength and basically stay home and keep fit and keep as well as they can for as long as they possibly can.... rather than deteriorating to a stage where they end up being shoved in a Nursing Home.” (Pod20) | |
| Personal and job satisfaction and achievement | “I think its satisfaction, knowing that you can... you’ve done something to make... you know, to try and improve someone’s life. Not really... it’s more of a feel good thing kind of thing.” (Pod13)  “I’m concerned, not just about their feet, that I’m concerned about their... you know, looking a bit more holistically, looking at certainly if they’re... if they lose some weight, it’s easier on their feet. And yeah, I’m sort of... they might see that I’m caring for them a bit better than if I wasn’t to mention and that sort for thing.” (Pod14)  “…why would you promote physical activity; why do I bother? You know so because it makes me feel good; because it’s part of my job?” (Pod15)  “Well I guess I just do it just to help people. I’m not really going to get anything – maybe it makes me feel a wee bit better, a little bit better you know.” (Pod15)  “I think that’s quite a positive role to play and I should think there’d be some actual benefit when that works, when you see people actually benefiting from that activity.” (Pod19)  “In terms of for me as a practitioner, probably the more that you provide advice in the area, the more feedback you get from patients and the more insight you get from patients as to what works and what doesn’t work for them. And I think that helps you as a practitioner.” (Pod2) | |
| Normative influences | | |
| Colleagues/other health professional | “…talking to other colleagues, other Podiatrists, Physiotherapists,…” (Pod11) | |
| **Effectiveness of promotion** | | |
| **Range of beliefs about efficacy** | | |
| Limited effect | *“I know a lot of people have... that I recommend exercise, who have taken up the exercise but never really talked about noticeable benefits.” (Pod1)“There wouldn’t be a huge number of people over the years that I’ve actually encouraged to start doing exercise.” (Pod8)* | |
| Follow up | *“I definitely see giving out the advice but the problem there is if you're not following up, not monitoring it then it often… from giving the advice you often probably stop doing it because you're not getting that feedback yourself in terms of "I'm giving this advice and have no idea whether people are taking it or implementing it.” (Pod12)* | |
| Positive effect | *“…somebody who maybe is chronically impaired or hasn’t really done a lot of exercise sometimes might go away and come back three months later and say, ‘you know what? It really made an impact on us and it was on my mind for a while and actually I have started it and I feel better.” (Pod4)* | |
| **Knowledge, education and skills** | | |
| **Lack of pre and post registration training** | *“…issues around health promotion and things would have come outside of undergraduate podiatry and I think there might have been some motivated, occasional lecturers that might have highlighted some issues, but it was by no means a formal part.” (Pod6)*  *“…it's not a particular subject at University - or it wasn't when I went through - but it's implied in so many areas.” (Pod10)* | |
| **Specific skills** | | |
| Lack of behavioural counselling | *“I guess it all comes back to things like behavioural change and it’s not an area which I’ve done any specific training,... I don’t feel as though I have the skills in behavioural change...” (Pod 2)* | |
| Lack of exercise program skills | *“I think that's where we start but certainly at this point in time I haven't got the skills or the knowledge to look at putting together some sort of exercise program.” (Pod9)* | |
| Motivational interview | *“I think I'd like to increase my skills in motivational interviewing so I would do some training in that, I think it's a good way to be able to ask people questions so they can… do that. I think chronic disease models are useful, they all have different ways of getting to the same ideas. (Pod13)* | |
